# Transgenerational effects of multiple mating in *Spodoptera litura* Fabricius (Lepidoptera: Noctuidae)

**DOI:** 10.1002/ece3.10189

**Published:** 2023-06-14

**Authors:** Xue‐yuan Di, Bin Yan, Jian‐feng Liu, Cheng‐xu Wu, Xiao‐fei Yu, Cecil L. Smith, Mao‐fa Yang

**Affiliations:** ^1^ Guizhou Provincial Key Laboratory for Agricultural Pest Management of the Mountainous Region, Scientific Observing and Experimental Station of Crop Pest in Guiyang, Institute of Entomology, Guizhou University Ministry of Agriculture Guiyang China; ^2^ College of Forestry Guizhou University Guiyang China; ^3^ College of Tobacco Science Guizhou University Guiyang China; ^4^ Georgia Museum of Natural History University of Georgia Athens Georgia USA

**Keywords:** multiple mating, offspring, *Spodoptera litura*, transgenerational effect

## Abstract

Polyandrous mating can result in sexual conflict and/or promote the evolution of mating patterns. Does multiple mating by females support the genetic benefits hypothesis and can it be validated as an evolutionary strategy? If we are to decipher the consequences of sexual interactions and understand the interplay of sexual conflict and multiple generational benefits, the transgenerational effects need to be followed over multiple generations. We investigated the effects of three mating patterns, single mating, repeated mating, and multiple mating, on parental *Spodoptera litura* copulation behavior, and then identified the impact on the development, survival, and fecundity of the F_1_ and F_2_ generations. Fecundity was not significantly affected in the F_1_ generation but was substantially enhanced in the F_2_ generation. There was a reversal of offspring fitness across the F_2_ generations from the F_1_ generations in progeny produced by multiple mating. In addition, the intrinsic rate of increase, finite rate of increase and net reproductive rate in the F_1_ generation the multiple mating treatment was significantly lower than in the single mating treatment, but there was no apparent effect on the F_2_ generation. Repeated mating had no significant effects on progeny fitness. We postulate that multiple mating imposes cross‐transgenerational effects and may ultimately influence multigenerational fitness in *S. litura.*

## Graphical abstract

Fecundity of *Spodoptera litura* multiple mating was not significantly affected in the F1 generation but was substantially enhanced in the F2 generation. There was a reversal of offspring fitness across the F2 generations from the F1 generations in progeny produced by multiple mating. We postulate that multiple mating imposes cross‐transgenerational effects and may ultimately influence multigenerational fitness in *S. litura*.

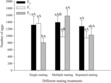

## 1 INTRODUCTION

Polyandry, which is widespread throughout many taxonomic groups, occurs when females mate with more than one male (Byrne & Whiting, 2011). However, females will often incur various costs of mating through increased risk of injury, disease, and predation (Ivy & Sakaluk, 2005). Nevertheless, the mating frequency of females often exceeds the number of copulations required for fertilization. In fact, a single mating is frequently sufficient for females to fertilize their entire complement of eggs and to maximize their reproductive success (Egan et al., 2016; Omkar & Sahu, 2012). Mating frequencies beyond that required for female fertilization can lead to sexual conflict (Backhouse et al., 2012). Conflict and competition between individuals have been shown to promote the evolution of varied mating patterns (Zhang et al., 2017). Widespread instances of polyandry have led to interest into the evolution of the behavior, especially in determining what the ecological and evolutionary consequences of polyandry might be (Boulton & Shuker, 2015).

Evolutionary explanations have suggested that females may gain direct and/or indirect benefits from polyandry (Havens et al., 2011; Moore et al., 2003; Tregenza et al., 2003; Xu et al., 2019). Repeated mating, which is repetitious mating with the same male mate, is a simple form of mate selection for polyandry (Jennions et al., 2007). In theory, females could obtain the same level of benefits via repeated mating with the same male as they derive from copulation with different males if sperm is not depleted (Sakaluk et al., 2002). Nevertheless, in many arthropod species including *Gryllus bimaculatus* DeGeer (Orthoptera: Gryllidae)*, Dermestes maculatus* (DeGeer) (Coleoptera: Dermestidae), and *Cordylochernes scorpioides* (L.) (Pseudoscorpionidae: Chernetidae), researchers have reported that females prefer to mate with new males when presented with a choice between males they have and have not mated with previously (Archer & Elgar, 1999; Bateman, 1998; Xu & Wang, 2009; Zeh et al., 1998).

Multiple mating may benefit females in instances where males transfer resources directly to the females during mating (Castrezana et al., 2017), such as replenishing sperm and nutrients in the ejaculate, which may result in an increased number of offspring (Egan et al., 2016; Hsu et al., 2014; Li et al., 2014; Xu et al., 2019). In addition, indirect benefits arise from the genetic benefit of increasing offspring genetic diversity (Dunn et al., 2009; Egan et al., 2016; Johnson & Brockmann, 2013; Tregenza & Wedell, 1998). Understanding the relative costs and benefits of repeated mating with a single male and multiple mating with several males is key to understanding the evolution of polyandry.

The process where mating patterns of the parental generation can greatly affect offspring developmental, and/or reproductive and evolutionary trajectories, is commonly referred to as transgenerational effects (Ducatez et al., 2012; Gao et al., 2020). The traditional view regarding polyandry states that there is a positive relationship between female mating frequency and the number of offspring produced. In addition, multiple mating by females is thought to increase the fitness of their offspring (McNamara et al., 2014), although there is a trade‐off with longevity (Kawazu et al., 2014). There is growing evidence to support this view: Although multiple mating in female *Anegleis cardoni* (Weise) (Coleoptera: Coccinellidae) increased their fecundity, their longevity decreased (Omkar & Sahu, 2012). Male offspring of the polyandrous vole *Myodes glareolus* (Schreber) (Rodentia, Cricetidae) and *Teleogryllus oceanicus* (Le Guillou) (Orthoptera: Gryllidae) are competitively superior to male offspring produced by monandrous parents (Kawazu et al., 2014; McNamara et al., 2014). However, offspring of other species, including *Cadra cautella* (Walker) (Lepidoptera: Pyralidae) and *Anaphes nitens* (Girault) (Hymenoptera: Mymaridae), do not appear to derive any benefit from female multiple matings (McNamara & Elgar, 2008; Santolamazza‐Carbone & Pestaña, 2010).

*Spodoptera litura* (Fabricius) (Lepidoptera: Noctuidae) is a serious worldwide agricultural and forest pest with high fecundity (Ahmad et al., 2007). Females of the species have multiple matings (Li et al., 2012; Wu et al., 2015), with paired insects mating as many as six times, with an average of 2.54 ± 0.33 matings in its lifetime (Di et al., 2020). In this study, we attempt to assess the benefit hypothesis of female polyandry in *S. litura* by measuring the effects resulting from single, multiple, and repeated matings of *S. litura* on the development and fitness of their offspring. We postulate that the mating pattern of females will affect the development and fecundity of their offspring. Studying the mating habits of this widespread species, while determining the plasticity of their offspring, is expected to provide a greater overall understanding of their ecology and evolution.

## 2 MATERIALS AND METHODS

### 2.1 Population maintenance and insect rearing

The colony of *S. litura* used in this study was established in June 2017 from individuals collected at Lanba Village, Majiang County, Guizhou Province, China (26°29′54.65″ N, 107°37′49.05″ E). The population was maintained in a climate chamber set at 27°C (±1), 60% (±5) relative humidity (RH) with a photoperiod of 14:10 h light/dark. Egg masses were collected and placed in Petri dishes (9 cm diameter) lined with moistened filter paper. Newly hatched neonates were reared en masse on fresh tobacco leaves in plastic cups (13 cm high, 17.8 cm diameter) until they reached the third instar. Around 30 third instars were transferred to ventilated transparent plastic containers (20.4 × 35.2 × 12.3 cm) until pupation. Larvae were provided with fresh tobacco leaves daily. One male and one female were paired and were kept in plastic cups (13 cm high, 17.8 cm diameter), and fed on 10% honey solution. Male and female pupae were separated based on external morphology and placed in separate plastic cups to prevent copulation.

### 2.2 Mating treatments of parental generation (F_0_ )

The females were assigned to either monandrous or polyandrous mating treatments. Three mating treatments were conducted. Treatment 1. Single mating: randomly assigned 1‐day‐old virgin females and males were paired. After a single mating, the male and female were separated. Treatment 2. Multiple matings: 1‐day‐old females were paired with a 1‐day‐old male. After mating once, the male was removed and replaced with a random virgin 1‐day‐old male and the female allowed to mate once with the second male and then separated. Treatment 3. Repeated matings: individual females were paired with the same male and allowed to mate twice and were then separated. Cotton balls soaked in a 10% honey solution were changed each day until adult death. Single mating, multiple mating, and repeated mating were mated 30, 20, and 28 times, respectively. Egg masses were collected and recorded daily until the death of the female. Egg masses from different treatments were transferred to separate Petri dishes and used for the experiments outlined below.

### 2.3 First generation (F_1_ ) life table after different mating treatments

In order to determine the effects of multiple and repeated matings on the offspring of *S. litura,* 100 randomly selected eggs were allocated to treatments the day after egg laying began obtained from each of the previously discussed mating treatments, when all mating for different mating treatments have been completed (single mating mated once, multiple mating, and repeated mating mated twice). Each group of 100 eggs were placed on moistened filter paper in plastic Petri dishes (2 cm high, 9 cm diameter). The newly hatched larvae were carefully transferred onto fresh tobacco leaves in individual 200 mL plastic vials (one per vial) (4 cm high, 8 cm diameter). The tobacco leaves were replaced as necessary, increasing in frequency with age until the larvae ceased feeding at the prepupal stage. Once offspring emerged as adults, females and males were paired at random and allowed to mate once. The females and males were then placed together in 1000 mL plastic containers and fed a 10% honey solution. Eggs were collected daily and placed on moistened filter paper in plastic Petri dishes as described above. Fecundity was evaluated by counting the number of eggs laid per female per day. The duration of each developmental stage and survival was assessed daily until the death of each individual. All insects in this and subsequent experiments were maintained in a climate chamber at 27 ± 1°C, 60 ± 5% RH and 14:10 h (L:D) photoperiod.

### 2.4 Second generation (F_2_ ) life table after different mating treatments

Based on first‐generation data obtained from the different mating systems, egg masses were collected from the three treatments after the F_1_ generation adults had mated (single mating, multiple mating, and repeated mating adults of the F_1_ generation all mate once). A total of 100 randomly collected eggs from each of the F_1_ generation mating treatments were collected and transferred to Petri dishes (2 cm high, 9 cm diameter) containing moistened filter paper, and allowed to hatch. All newly hatched larvae were then individually transferred into 200 mL plastic vials (4 cm high, 8 cm diameter) and provided with tobacco leaves. The tobacco leaves were changed daily to maintain adequate nutrition until pupation. After emergence, adults from the same mating treatment were paired and allowed to mate once. Eggs were collected each day. The developmental, survival, longevity, and reproductive data were recorded as described above until the death of all individuals.

### 2.5 Data analysis

The raw data for copulation duration, number of eggs, and longevity of the F_0_, F_1,_ and F_2_ generation individuals were analyzed using SPSS 21 (SPSS 21.0, IBM). All data were tested for normality using the Kolmogorov–Smirnov test and met the assumption of ANOVA. The number of eggs’ data was transformed into a logarithmic transformation to approximate a normal distribution. The number of eggs and longevity were subsequently analyzed by one‐way analysis of variance (ANOVA) followed by Tukey's HSD test (*p* < .05). The distribution of the copulation duration data was skewed and was analyzed by nonparametric Kruskal–Wallis analysis of variance. All data are presented as the mean ± standard error (SE). Survival curve analysis was evaluated by log‐rank test for the different mating treatments. The parameters of intrinsic rates of increase, finite rate of increase, net reproductive rates, and mean generation time were calculated using the bootstrap method included in the computer program TIMING–MSChart (Chi, 2020). Because bootstrap analysis uses random resampling, a small number of replications will generate variable means and standard errors. To generate fewer variable results, 100,000 replications were used in this study.

## 3 RESULTS

### 3.1 Effects of mating treatment on parental generation (F_0_ )

**FIGURE 1:**
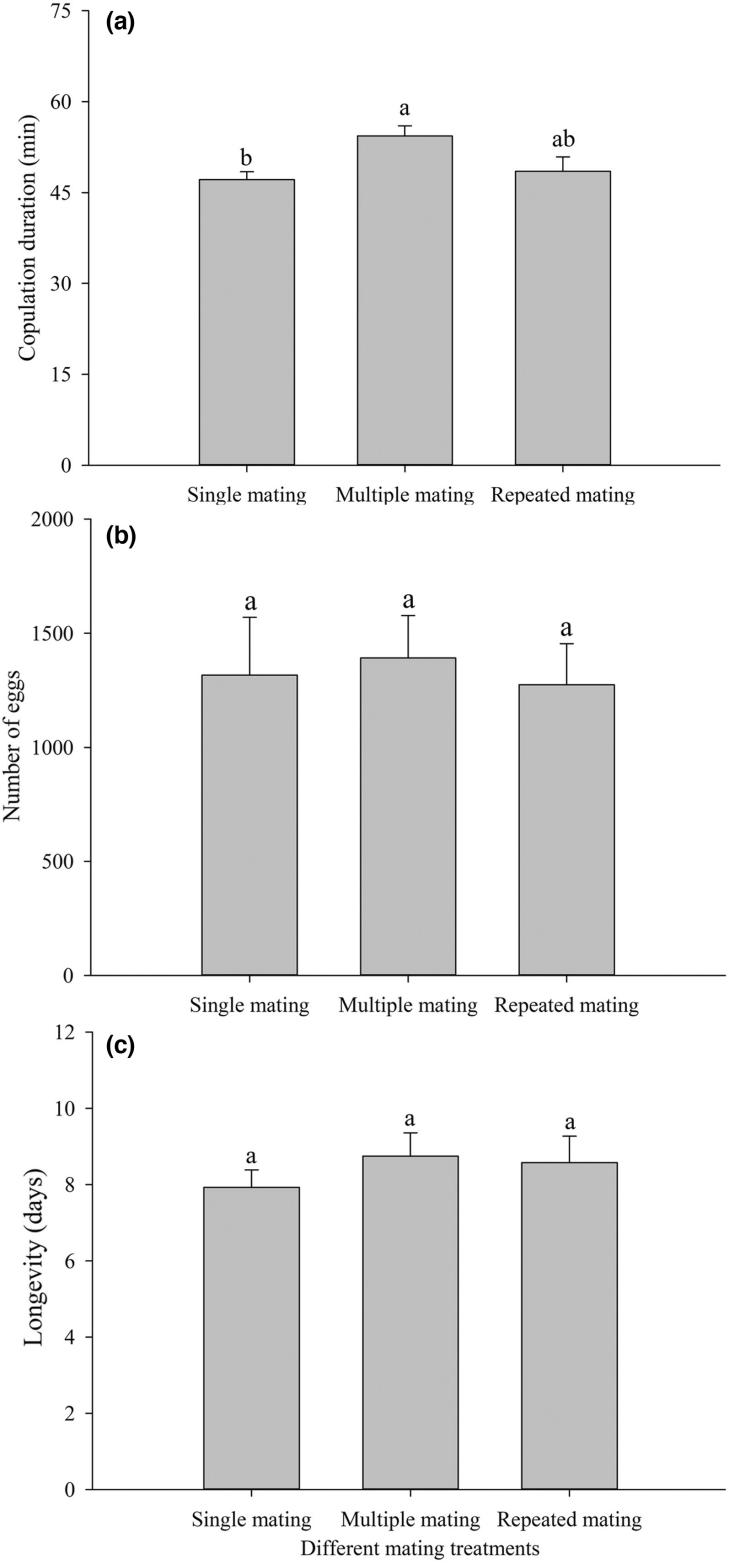
Copulation duration (a), number of eggs (b), and longevity of females (c) of F_0_ generation of *Spodoptera litura* in different mating treatments. Different lowercase letters on the top of the bars indicate the significant differences among different mating treatments. Multiple and repeated mating are only analyses of the second mating duration in (a).

Multiple mating did not significantly affect the number of eggs (*F*_2,61_ = 0.273, *p* = .762) or the longevity (*F*_2,84_ = 0.619, *p* = .541) of the females, but did significantly affect the copulation duration (*df* = 2, χ^2^ = 11.849, *p* = .003; Figure 1). There was no significant difference in the copulation duration between single mating and repeated mating of different mating treatments, but there was a significant difference in the copulation duration between single mating and multiple mating of different mating treatments. The single mating treatment was noticeably shorter than the multiple mating treatment.

### 3.2 Transgenerational effect on immature developmental time of offspring

**FIGURE 2:**
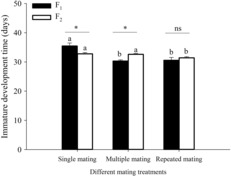
Immature development time of *Spodoptera litura* F_1_ and F_2_ generations in different mating treatments. Different lowercase letters on the top of bars indicate significant differences among different mating treatments. Asterisks indicate differences between different generations.

The immature development time in different mating treatments and different generations was markedly different (Figure 2). In the F_1_ generation, the development time of immatures from single matings was noticeably longer than that of the multiple mating and repeated mating treatments. In the F_2_ generation, however, the development time of immatures in the repeated mating treatment was significantly shorter than in the single mating treatment and multiple mating treatment. The development time of immatures in the single mating F_1_ generation was significantly longer than it was in the F_2_ generation, but multiple mating and repeated mating in the F_1_ generation was significantly shorter than in the F_2_ generation.

### 3.3 Transgenerational effect on fecundity of offspring

**FIGURE 3:**
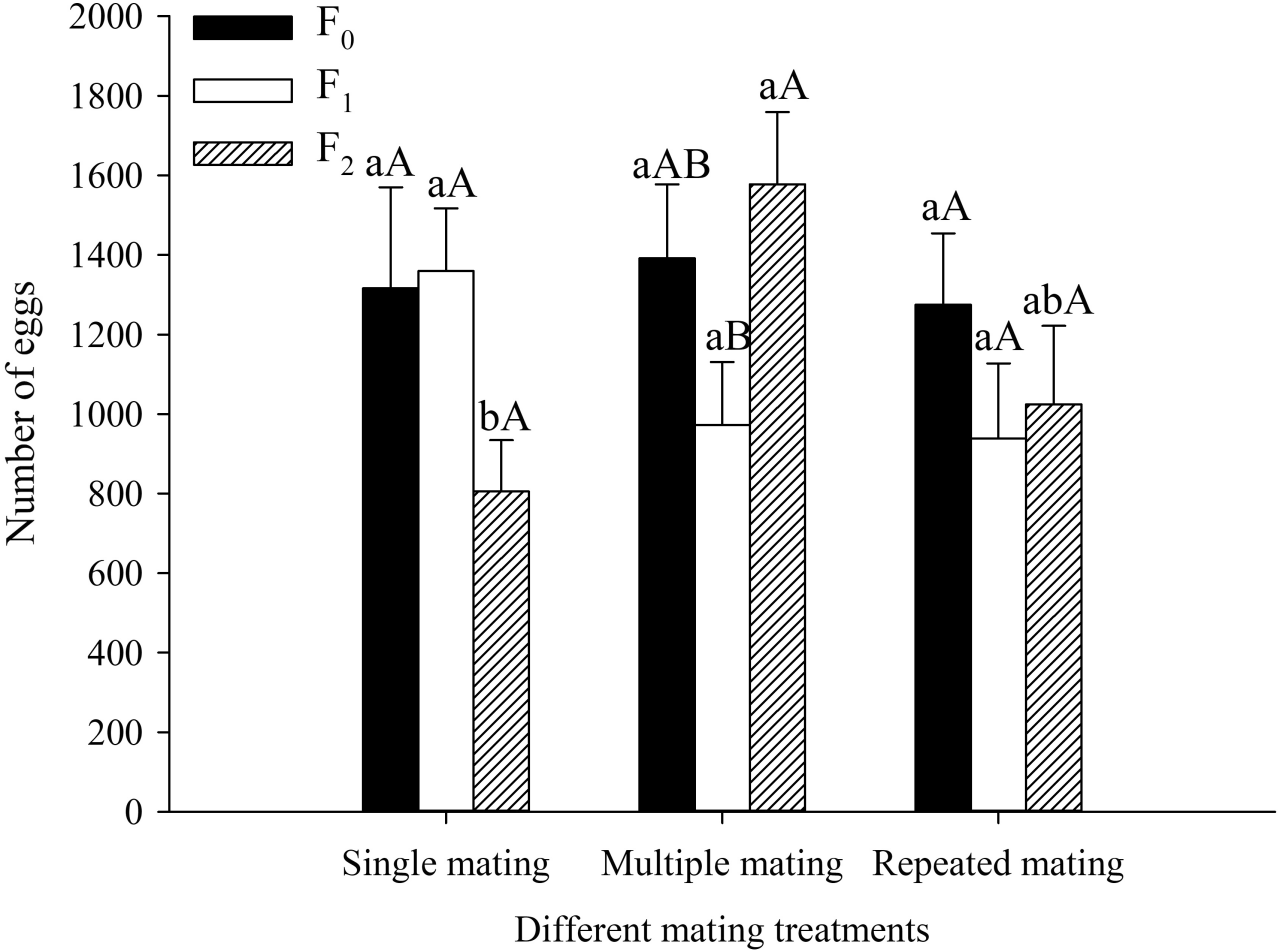
Number of eggs produced by *Spodoptera litura* F_0_, F_1_ and F_2_ generations after different mating treatments. Different lowercase letters on the top of the bars indicate the significant differences among different mating treatments at *p* < .05 by Tukey's HSD multiple range test. Different uppercase letters on the top of the bars indicate the significant differences among different generations at *p* < .05 by Tukey's HSD multiple range test.

Significant differences occurred in the fecundity in different generations and in different mating treatments (Figure 3). There was no significant difference in the number of eggs among the different mating treatments (F_0_: *F*_2,61_ = 0.084, *p* = .920; F_1_: *F*_2,85_ = 1.942, *p* = .150). The number of offspring in the F_2_ generation of the multiple matings, however, was significantly higher than in the single and repeated matings (*F*_2,55_ = 6.351, *p* = .003). In multiple matings, egg production was significantly greater in the F_2_ generation than in the F_1_ generation (*F*_2,67_ = 3.267, *p* = .044). There was no significant difference in reproduction in different generations between single mating and repeated mating (single mating: *F*_2,87_ = 2.534, *p* = .085; repeated mating: *F*_2,74_ = 0.939, *p* = .398).

### 3.4 Transgenerational effect on survival rate

**FIGURE 4:**
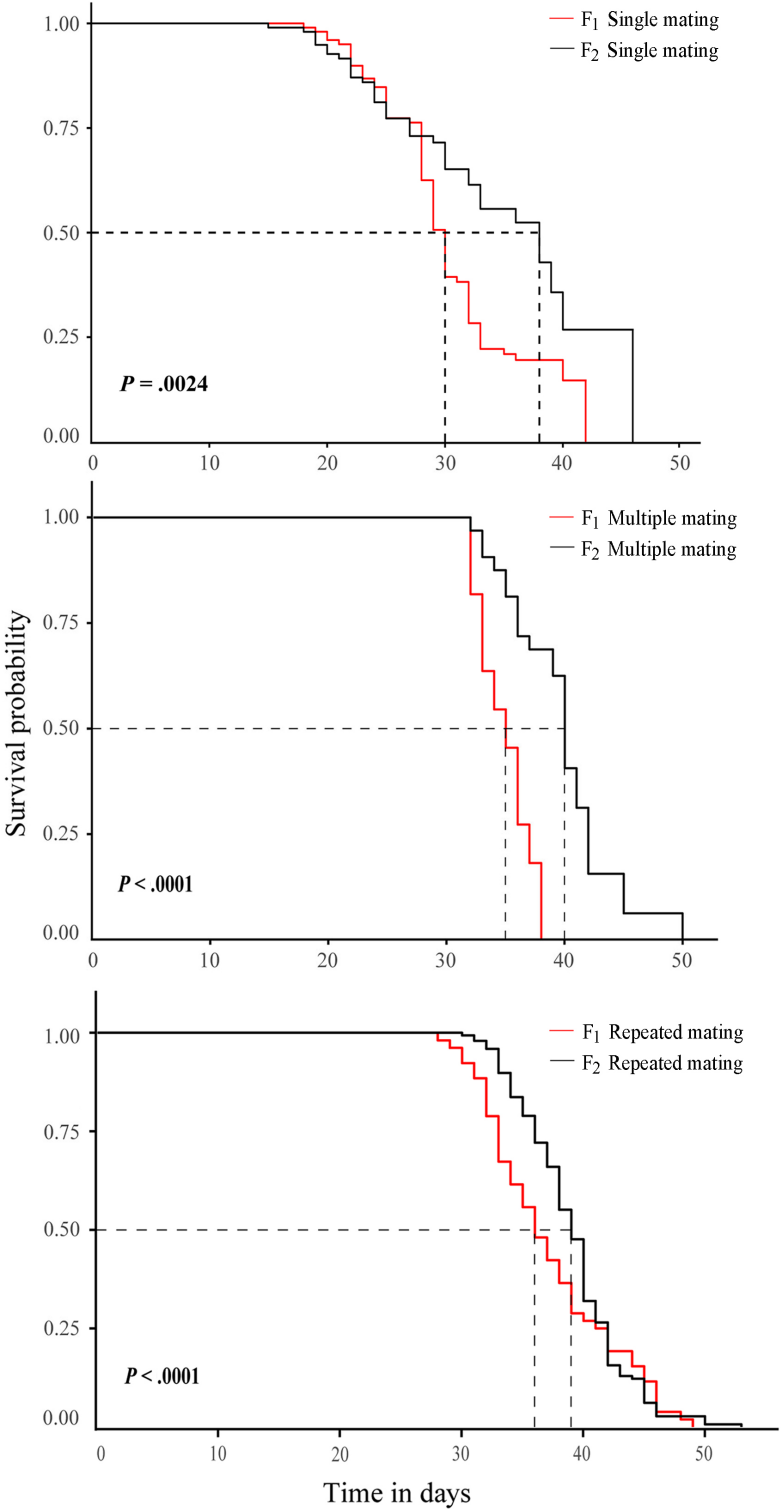
Survival probability of *Spodoptera litura* F_1_ and F_2_ generations after different mating treatments.

The survival rates were significantly different in the different generations within different mating treatments (Figure 4). The survival rates of the F_2_ generation, overall, were higher than those of the F_1_ generation.

### 3.5 Transgenerational effect on longevity

**FIGURE 5:**
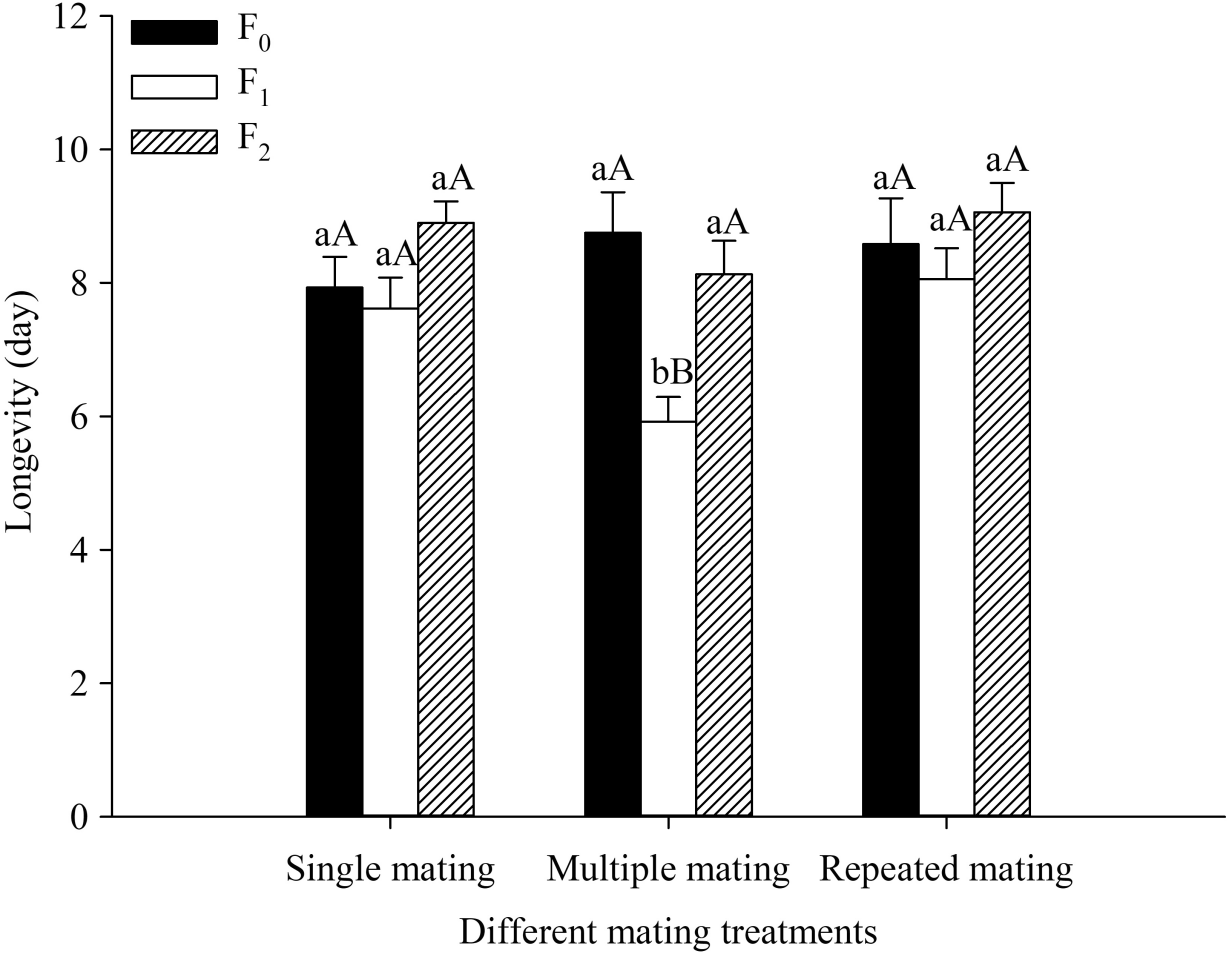
Longevity of *Spodoptera litura* F_0_, F_1,_ and F_2_ generations in different mating treatments. Different lowercase letters on the top of bars indicate significant differences among different mating treatments at *p* < .05 by Tukey's HSD multiple range test. Different uppercase letters on the top of bars indicate significant differences among different generations at *p* < .05 by Tukey's HSD multiple range test.

There was a significant difference in the longevity from the different mating treatments (Figure 5). There was no significant difference between the longevity of the F_0_ and F_2_ generations (F_0_ generation: *F*_2,125_ = 2.897, *p* = .059; F_2_ generation: *F*_2,92_ = 0.684, *p* = .507). In the F_1_ generation the longevity of multiple mating was significantly lower than it was in individuals from single mating and repeated mating (*F*_2,60_ = 5.220, *p* = .008). There was no significant difference in longevity between single mating and repeated mating in the F_0_, F_1,_ and F_2_ generations (Single mating: *F*_2,87_ = 2.534, *p* = .085; Multiple mating: *F*_2,74_ = 0.939, *p* = .398). The longevity of F_1_ generation from multiple matings was significantly shorter than F_0_ and F_2_ generation (*F*_2,67_ = 3.267, *p* = .044).

### 3.6 Transgenerational effect on population parameters

The intrinsic rate of increase (*r*), finite rate of increase (*λ*), net reproductive rate (*R*_0_), and mean generation time (*T*) for the different treatments and generations are listed in Table 1. Significant differences were observed in the population parameters of the offspring from different maternal mating treatments in the F_1_ and F_2_ generations. In the F_1_ generation, the values for the intrinsic rate of increase and finite rate of increase in the single mating treatment (*r =* 0.138 d^−1^, *λ* = 1.149 d^−1^) were higher than those in the multiple mating treatment (*r =* 0.087 d^−1^, *λ* = 1.091 d^−1^). These values, however, were significantly higher in the repeated mating treatment (*r =* 0.176 d^−1^, *λ* = 1.168 d^−1^) of the F_2_ generation. There were significant differences in the net reproductive rate of the F_1_ generation and mean generation time of the F_2_ generation.

**TABLE 1:** Population parameters (mean ± SE) of the F_1_ and F_2_ generations of *Spodoptera litura* after different mating treatments.

Population parameter	Generation	Single mating	Multiple mating	Repeat mating
*r* (Intrinsic rate of increase) (d^−1^)	F_1_	0.138 ± 0.012aA	0.087 ± 0.019bB	0.124 ± 0.013abB
	F_2_	0.154 ± 0.008bA	0.155 ± 0.008bA	0.176 ± 0.007aA
*λ* (Finite rate of increase) (d^−1^)	F_1_	1.149 ± 0.014aA	1.091 ± 0.020bB	1.132 ± 0.0151abA
	F_2_	1.166 ± 0.009bA	1.168 ± 0.010bA	1.192 ± 0.008aB
*R*_0_ (Net reproductive rate) (offspring per individual)	F_1_	130.75 ± 46.355aA	21.29 ± 11.615bB	71.53 ± 28.380abB
	F_2_	199.14 ± 46.261aA	252.34 ± 72.316aA	316.19 ± 61.571aA
*T* (Mean generation time) (day)	F_1_	35.086 ± 0.930aA	35.196 ± 0.491aA	34.388 ± 1.321aA
	F_2_	34.383 ± 0.608aA	35.609 ± 0.341aA	32.698 ± 0.390bA

Data in the table are means ± SE. Means followed by different lowercase letters in the same row are significantly different among mating treatments. Means followed by different uppercase letters in the columns row are significantly different between generations. Standard errors were estimated by using 100,000 bootstrap resampling.

## 4 DISCUSSION

According to Arnqvist and Nilsson (2000), understanding mating behavior in insects is at the heart of comprehending their ecology and evolution. The sexy‐sperm and bet‐hedging hypothesis put forth by several researchers suggest that polyandry is likely to be an evolutionary strategy (Egan et al., 2016; McNamara et al., 2014; Sarhan & Kokko, 2007). Polyandry is a common phenomenon in many lepidopteran species (Jennions & Petrie, 2000; Kawazu et al., 2014; Royer & McNeil, 1993; Taylor et al., 2014; Wiklund et al., 2001). In order to thoroughly understand the effectiveness of this mating behavior, it is essential to follow the fitness gained (or lost) through successive generations (Zajitschek et al., 2018). In this study, transgenerational fitness was observed after different mating treatments of *S. litura*. Maternal mating had no significant effect on the F_0_ and F_1_ generations, but clearly increased fecundity in the F_2_ generation.

Polyandry is not only an evident mechanism for supporting genetic heterogeneity but also a behavior that affects the adaptation of females and their progeny (Ryazanova, 2011; Taylor et al., 2014). Its benefits may manifest more in the subsequent mating success of offspring than in their immediate viability (Sakaluk et al., 2002). Several previous studies have noted that in some species, multiple mating could increase offspring fecundity, fitness, and reproductive success (Bernasconi & Keller, 2001; Byrne & Whiting, 2011; Havens et al., 2011; Ivy & Sakaluk, 2005; Jennions & Petrie, 2000; McLain, 1998; Omkar & Sahu, 2012). In *Anegleis cardoni* (Weise) (Coleoptera: Coccinellidae) and *Cnaphalocrocis medinalis* (Guenée) (Lepidoptera: Crambidae), polyandry may provide direct benefits to females, generating mating gifts and increasing fecundity (Kawazu et al., 2014; Omkar & Sahu, 2012; Worthington & Kelly, 2016). In others, however, offspring fitness was not markedly different (House et al., 2011; Klemme et al., 2014; Zajitschek et al., 2018). The *S. litura* is polygynandrous (Di et al., 2020). However, previous studies failed to indicate whether multiple mating by female *S. litura* affected reproduction and longevity in their maternal generation (Bezzerides et al., 2008; Klemme et al., 2014). No evidence was found for any short‐term parental benefits resulting from *S. litura* polyandry. Our results demonstrated that multiple mating increases offspring fecundity. Multiple mating had no significant effect on the fecundity and longevity in F_0_ generation, but significantly increase fecundity of F_2_ generation. Therefore, multiple mating may bring fitness benefit on fecundity of *S. litura*.

Although several researchers have suggested that multiple mating could increase genetic benefits in the form of genetic diversification and increase genetic compatibility (Bilde et al., 2009; Boulton & Shuker, 2015; Johnson & Brockmann, 2013), others have found that repeated mating seems to be beneficial solely in terms of fecundity (Ivy & Sakaluk, 2005; Omkar & Mishra, 2005; Walker & Allen, 2010). Interestingly, the females of many insect species appear to discriminate against previous mates (Archer & Elgar, 1999; Bateman, 1998; Xu & Wang, 2009; Zeh et al., 1998). Male mating history may also have an effect on the mating choices in some females (Michaud et al., 2013). *S. litura* females have been shown to have a preference for their previous mates over novel males (Li et al., 2014). Moreover, in *Nicrophorus vespilloides* (Herbst) (Coleoptera: Silphidae) and the cricket, *G. bimaculatus* De Geer repeated mating apparently confers no known indirect benefits to their offspring (House et al., 2008; Tregenza & Wedell, 1998). Our results demonstrated that repeated mating had no obvious effects on fecundity compared with single mating. A possible explanation for this would be that repeated disturbance could affect fecundity (Li et al., 2015). Moreover, repeated mating could be costly to female *S. litura* in terms of time and energy.

Sexual interactions experienced by females in one generation can potentially permeate through subsequent generations, affecting the reproductive success and survival of future generations (Zajitschek et al., 2018), that is, transgenerational effects (Heard & Martienssen, 2014). It is necessary to evaluate fitness consequences over successive generations if we want to understand the consequences of sexual interactions. The results of our study indicate that as a consequence of multiple mating there was a reversal of offspring fitness in the F_2_ generations, where fecundity in the F_1_ generations was not significantly increased, but was substantially enhanced in the F_2_ generations. We suspect that multiple mating of *S. litura* not only induces cross‐generational fitness, but, in general, is a behavior that is beneficial to the fecundity of *S. litura*. The multiple mating confers transgenerational benefits, which is in line with Zajitschek et al. (2018). Reversal effects in progeny fitness across different generations have also been found in *Drosophila melanogaster*, where the fitness of the sons increased but grandsons decreased with increasing maternal sexual interactions (Brommer et al., 2012). Therefore, even in the absence of immediate benefits due to multiple mating, it will be necessary to determine whether multiple mating behavior has a potential reversing effect that is retained in succeeding generations as well, because transgenerational effects resulting from polyandry may potentially influence the rate and extent of evolutionary change (Bloch Qazi et al., 2017; Bonduriansky & Day, 2009). However, it is still unclear whether polyandry influences only the F_2_ generations or if it enables effects that may extend into ensuing generations. This will need further study.

## 5 CONCLUSION

Bet‐hedging theory explains how a group of individuals should optimize fitness in unpredictable and varying environments by sacrificing the mean fitness in order to decrease variation in fitness (Olofesson et al., 2009). Life history and mating behavior can be modulated by conditions experienced by a parental generation, and such transgenerational effects may arise from epigenetic mechanisms or “bet‐hedging” (Henshaw & Holman, 2015; Tougeron et al., 2020; Zajitschek et al., 2018). This study demonstrates that multiple mating by *S. litura* females caused cross‐transgenerational effects. Although there were no discernable effects on the F_1_ generations, significantly improvements were seen in the fecundity of the F_2_ generations. Indeed, maternal polyandry had effects that carried over for at least two generations. Our study highlights the importance of continuing observations of the effects across multiple generations to fully comprehend the net transgenerational consequences of sexual interactions. Even when there are no observable immediate costs or benefits due to sexual conflict, potential reversal effects in subsequent generations need to be considered to reveal the stability and mechanisms of transgenerational effects and long‐term consequences that may result from the evolution of diverse mating patterns.

## AUTHOR CONTRIBUTIONS

**Xue‐yuan Di:** Conceptualization (equal); investigation (equal); writing – original draft (equal). **Bin Yan:** Investigation (equal); methodology (equal). **Jian‐feng Liu:** Supervision (equal); writing – review and editing (equal). **Cheng‐xu Wu:** Formal analysis (equal). **Xiaofei Yu:** Supervision (equal). **Cecil L Smith:** Writing – review and editing (equal). **Mao‐fa Yang:** Project administration (equal); writing – review and editing (equal).

## FUNDING INFORMATION

This research was supported by the National Natural Science Foundation of China (grant No. 31960540), the Science and Technology Project of Guangxi Zhuang Autonomous Region Branch Company of China Tobacco Company (grant No. 2021–02), and the Guizhou Province Science and Technology Innovation Talent Team Project (grant No. Qian Ke He Pingtai Rencai–CXTD [2021]004).

## CONFLICT OF INTEREST STATEMENT

The authors declare that they have no conflicts of interest.

## Data Availability

All data are available on FigShare (DOI: https://doi.org/10.6084/m9.figshare.22811912).
